# Bioaccumulation of Cry1Ab Protein from an Herbivore Reduces Anti-Oxidant Enzyme Activities in Two Spider Species

**DOI:** 10.1371/journal.pone.0084724

**Published:** 2014-01-13

**Authors:** Ji Zhou, Kaifu Xiao, Baoyang Wei, Zhi Wang, Yun Tian, Yixing Tian, Qisheng Song

**Affiliations:** 1 College of Bioscience & Biotechnology, Hunan Agriculture University, Changsha, China; 2 Division of Plant Sciences, University of Missouri, Columbia, Missouri, United States of America; CINVESTAV-IPN, Mexico

## Abstract

Cry proteins are expressed in rice lines for lepidopteran pest control. These proteins can be transferred from transgenic rice plants to non-target arthropods, including planthoppers and then to a predatory spider. Movement of Cry proteins through food webs may reduce fitness of non-target arthropods, although recent publications indicated no serious changes in non-target populations. Nonetheless, Cry protein intoxication influences gene expression in Cry-sensitive insects. We posed the hypothesis that Cry protein intoxication influences enzyme activities in spiders acting in tri-trophic food webs. Here we report on the outcomes of experiments designed to test our hypothesis with two spider species. We demonstrated that the movement of CryAb protein from *Drosophila* culture medium into fruit flies maintained on the CryAb containing medium and from the flies to the spiders *Ummeliata insecticeps* and *Pardosa pseudoannulata*. We also show that the activities of three key metabolic enzymes, acetylcholine esterase (AchE), glutathione peroxidase (GSH-Px), and superoxide dismutase (SOD) were significantly influenced in the spiders after feeding on Cry1Ab-containing fruit flies. We infer from these data that Cry proteins originating in transgenic crops impacts non-target arthropods at the physiological and biochemical levels, which may be one mechanism of Cry protein-related reductions in fitness of non-target beneficial predators.

## Introduction

Transgenic rice lines expressing Cry protein-encoding genes from the bacterium *Bacillus thuringiensis* (*Bt*) have recently been developed to control the major lepidopteran pests, the stem borers and leaffolders in China [1], in wake of environmental contamination and pest outbreak due to the continuous use and overuse of broad spectrum pesticides over the past several decades [2]. Despite the development of at least 8 transgenic rice lines expressing toxin [3], [4], [5], [6], [7], [8], [9], no *Bt* rice lines have yet been approved for commercial release in China. However, one cultivar (Huahui 1) and its hybrid line (Shanyou 63), expressing Cry1Ab and Cry1Ac for resistance to lepidoperans, have been granted a limited trial in farmlands in Hubei Province for the five-year period 2009 through 2014 [10].

While transgenic rice offers tremendous benefits to agricultural production and the environment, substantial risks are associated with the deployment of *Bt* rice lines. One concern is the potential movements of *Bt* toxins through natural food webs, including non-target species, in rice cropping systems. Bernal et al. (2002) investigated five transgenic rice lines for movement of Cry proteins from rice to brown planthoppers (non-target herbivore) and to its predator, the plant bug, *Cyrtorhinus lividipennis* [11]. While they detected the Cry proteins in the planthopper honeydew and in the predator, they concluded that exposure to *Bt* proteins might not influence the fitness of either insect species. Li et al. (2007) came to a similar conclusion based on the outcomes of a three-year study of arthropod communities in rice paddy fields [12]. They reported substantial similarity between arthropod communities in experimental *Bt* rice crops and control *non-Bt* rice crops. Another field study, using three sites in Zhejiang Province, considered three planthopper species and their predator, *C. lividipennis* [13]. Again, their two-year program recorded no differences in plant hopper populations, with *Sogatella furcife*a remaining the predominant planthopper in experimental and control plots. The *Bt* rice was not deleterious to the predator populations and the authors inferred that *Bt* rice may control the targeted lepidopteran pests while not influencing an important non-target species, the planthopper biocontrol agent, *C. lividipennis*. With respect to planthoppers, Chen et al. (2003a) reported that the transgenic lines TT9-3 and TT9-4, expressing a fused Cry1Ab-Cry1Ac gene, reduced brown planthopper feeding and oviposition behaviors, relative to the non-transgenic parental line, IR72 [14]. They inferred that the transgenic rice lines reduced planthopper fitness. Similarly, they reported that transgenic rice lines influenced dispersal of planthoppers and leafhoppers in field conditions [15].

Cry proteins are certainly transferred from rice plants to planthoppers, *Nilaparvata lugens*, and on to one of its predators, the wolf spider, *Pirata subpiraticus* [16]. Spiders are generalist predators and the idea that spiders can reduce populations of pest insects is well established [17], [18]. *Pardosa pseudoannulat*a and *Ummeliata insecticeps* are the dominant spider species in Chinese farmlands. Tian et al. (2012) demonstrated in field trials the movement of a *Bt* protoxin, Cry1Ab, from the *Bt* rice lines KMD1 and KMD2 through the brown planthopper, *N. lugens* to the ground spider *P. pseudoannulata* and showed that the Cry1Ab expressing rice did not influence the spider's fitness as determined by several parameters [19]. Alternatively, Chen et al. (2003b) suggested that *Bt* rice lines reduced the fitness of at least some non-target species, raising questions on the sub-lethal impacts of *Bt* rice at the level of physiology and biochemistry [15]. Oppert et al. (2012) reported on changes in transcriptome expression during *Bt* intoxication in larvae of Cry3Aa-sensitive beetles, *Tenebrio molitor* [20]. Among many changes in transcript expression, they reported down-regulation of transcripts encoding antioxidant enzymes, including superoxide dismutase, glutathione S transferase and catalase. We posed the hypothesis that the dietary ingestion of *Bt* protein could also influence enzyme activities in spiders acting in tri-trophic food webs. Here we report on the outcomes of experiments designed to test our hypothesis, using the spiders *P. pseudoannulata* and *U. insecticeps* as the test animals.

## Materials and Methods

### Drosophila processing

Routine *Drosophila* culture medium consists of cane sugar (124 g), corn starch (165 g), agar (124 g), and yeast powder (2 g) dissolved in 1 liter distilled water. The routine (or control) medium is prepared by mixing the components, boiling the mixture, cooling it down to 60°C and finally transferring aliquots of selected size to culture bottles. Experimental medium containing a final concentration Cry1Ab at 100 ng/ml (a predetermined effective dose to ensure sufficient *Bt* protein in medium for development of each fly from larva to adult in the bottle with about 300–600 flies), was prepared by adding the Cry1Ab protein (purchased from Shanghai YouLong Biotechnology Co.) into the routine medium after the temperature of the newly prepared medium was below 50°C.

*Drosophila melanogaster* (obtained from the genetics laboratory, Hunan Agricultural University) was reared on control or experimental media for the indicated times as noted in Results. To analyze movement of Cry1Ab protein from flies to spiders, separate groups of control and experimental flies were maintained on control or experimental media for 10 days, and then used in spider feeding experiments.

### Spider cultures

*P. pseudoannulata* and *U. insecticeps* were collected from the experimental farmland in Hunan Academy of Agricultural Sciences. No specific permissions were required for a limited number of spiders collected from this location because the sample collection did not involve endangered or protected species. Individual spiders (both species) were held in glass tubes (12×100 mm) maintained in the laboratory at room temperature and natural photoperiod. The spiders were provided moist cotton balls for water and supplied with 14 control or experimental flies per day.

### Estimating quantities of Cry1Ab protein in flies and spiders

Cry1Ab quantities in flies and spiders were determined using the Bt-Cry1Ab/Ac ELISA kits (American EnviroLogix, Inc., Portland, ME USA, distributed by Shanghai YouLong Biotech Co.). After recording wet weights, spiders (13) and flies (26) were homogenized in 1 ml PBS buffer using glass homogenizers (2 ml). The homogenates were centrifuged at 16,000*g* for 10 min at 4°C. One aliquot (0.25 ml) of each supernatant was used to determine protein concentrations via the Bradford assay (Bradford, 1976) [21] against BSA as a quantitative standard and a second aliquot (0.1 ml) was used to estimate Cry1Ab protein concentrations. Each assay used 26 flies and 13 mature spiders. Three biological replicated were performed for each assay.

### Enzyme assays

Spiders were maintained on experimental or control flies for 3 to 11 days. Spiders (13/treatment) were homogenized in 1 ml PBS then centrifuged at 16,000 g for 10 min at 4°C. Several aliquots were taken from the resulting supernatant. One aliquot (0.25 ml) was used for protein determination, another (0.1 ml) for Cry1Ab determination, a third (0.1 ml) to assay acetylcholine esterase (AChE; EC 3.1.1.7) activity following the method of Gorun (1978) [22], a fourth to assay glutathioine peroxidase (GSH-Px; EC 1.11.1.9) activity, and a final aliquot to assay superoxide dismutase (SOD; EC 1.15.1.1) activity. GSH-Px and SOD activities were determined using the commercial enzyme activities kits (Chinese Nanjing Jiancheng Bioengineering Institute, Nanjing, China). Three biological replicated were performed for each assay.

### Data analysis

The data were analyzed using the SPSS 19.0 version of Wilcoxon signed-rank test. Significant differences at p<0.05 or p<0.01 were designated with * or ** respectively.

## Results

### Cry1Ab protoxin moved from the fruit fly culture medium to fruit flies and spiders

**Figure 1:**
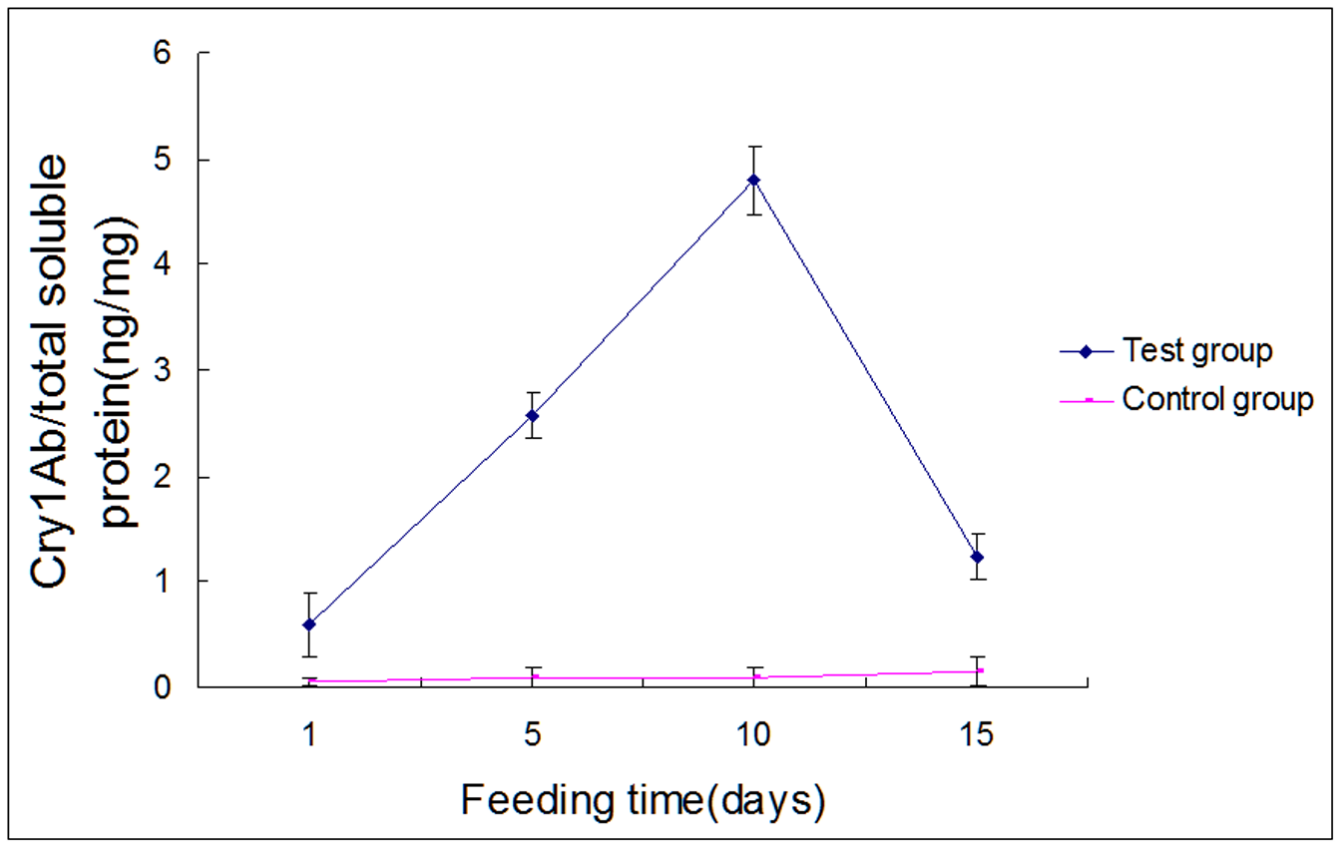
Cry1Ab protein concentrations in *D. melanogaster* at the indicated feeding times. The data points represent the mean ± SE of three independent biological replicates. All points representing experimental fruit flies are significantly different from points representing controls.

Figure 1 displays the accumulation and clearance of the Cry1Ab protein by fruit fly tissues. The Cry1Ab protein was not detected in fruit flies reared on control media. Flies reared on Cry1Ab-supplemented diets accumulated the Cry1Ab protein over the experimental feeding regimen, from about 0.6 ng Cry1Ab/mg protein on day 1 to almost 5 ng Cry1Ab/mg protein on day 10. Cry1Ab protein concentrations declined to just over 1 ng/mg by day 15.

**Figure 2:**
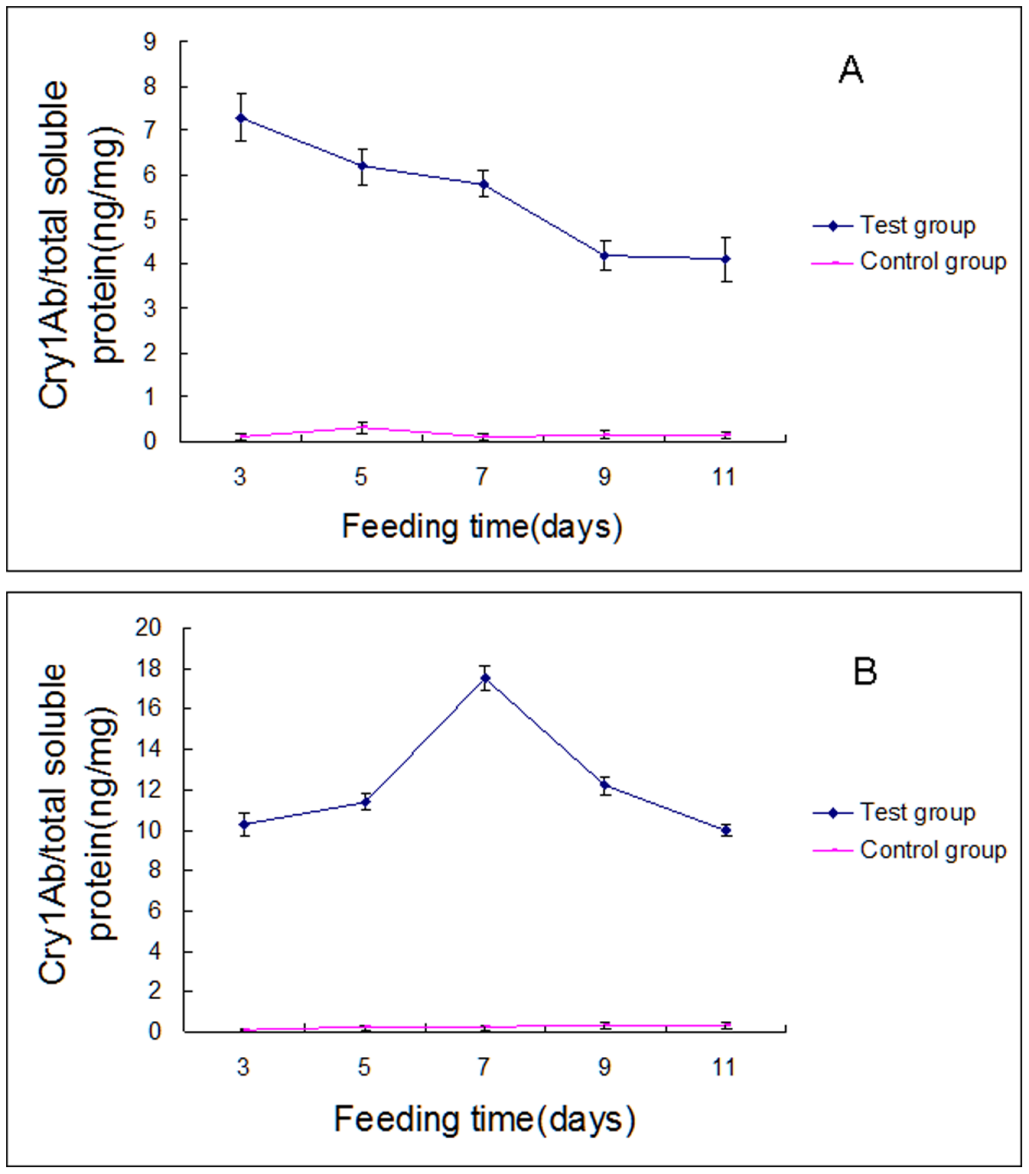
Cry1Ab protein concentrations in, Panel A, *U. insecticeps* and, Panel B, *P. pseudoannulata* at the indicated times. The data points represent the mean ± SE of three biological replicates. All points representing experimental spiders are significantly different from points representing controls.

Figure 2 shows that both spider species accumulated the Cry1Ab protein from fruit flies maintained on experimental culture media, although the patterns of accumulation differed between the two species. *U. insecticeps* accumulated Cry1Ab protein at about 7 ng/mg on day 3, then declined steadily over the following six days to about 4 ng/mg and remained constant over the next two days. The pattern of Cry1Ab accumulation was different in *P. pseudoannulata* (Fig. 2B) and these spiders accumulated about 10 ng Cry1Ab/mg protein after three days feeding on experimental fruit flies, increased to about 18 ng/mg protein by day 7, and then declined over the next four days to about the day 3 value.

### Dietary Cry1Ab influenced enzyme activities in spiders fed on experimental fruit flies

**Figure 3:**
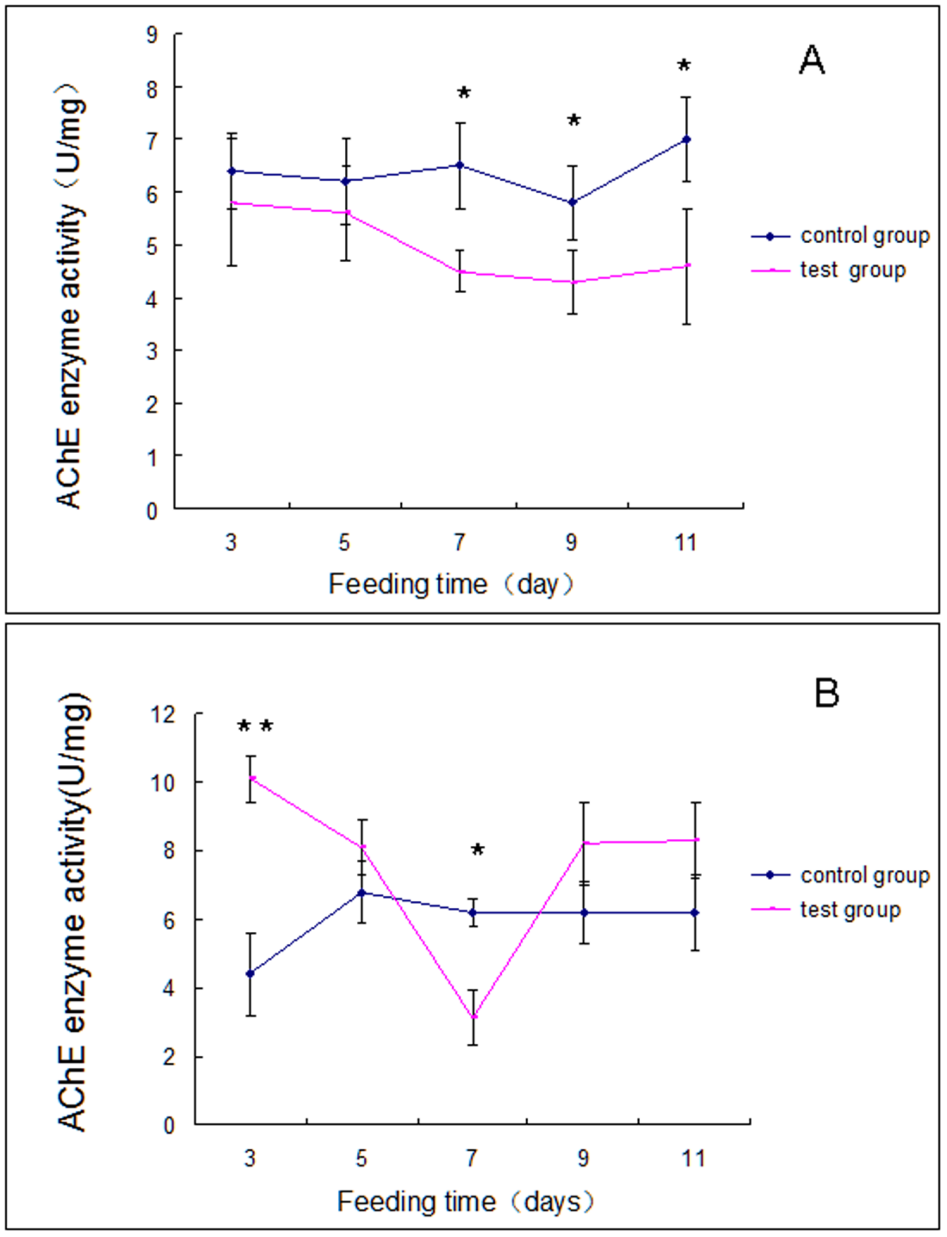
AChE activity in, Panel A, *U. insecticeps* and, Panel B, *P. pseudoannulata* at the indicated time periods. Each data point reflects the mean ± SE of three biological replicates. The significant differences (*p*<0.05) are indicated by an asterisk. The most significant differences (*p*<0.01) are indicated by two asterisks.

Fruit fly-derived Cry1Ab protein did not influence AChE activity in *U. insectice*ps until day 7 of the experimental feeding regimen. For days 7, 9 and 11, AChE activity was reduced, relative to controls, in the experimental spiders (Fig. 3A). Again, the pattern differed for *P. pseudoannulat*a, in which AChE activity was approximate 2-fold higher in experimental spiders compared to controls on day 3, then significantly lower in the experimentals by day 7. AChE activities did not differ between the experimentals and controls on the other days.

**Figure 4:**
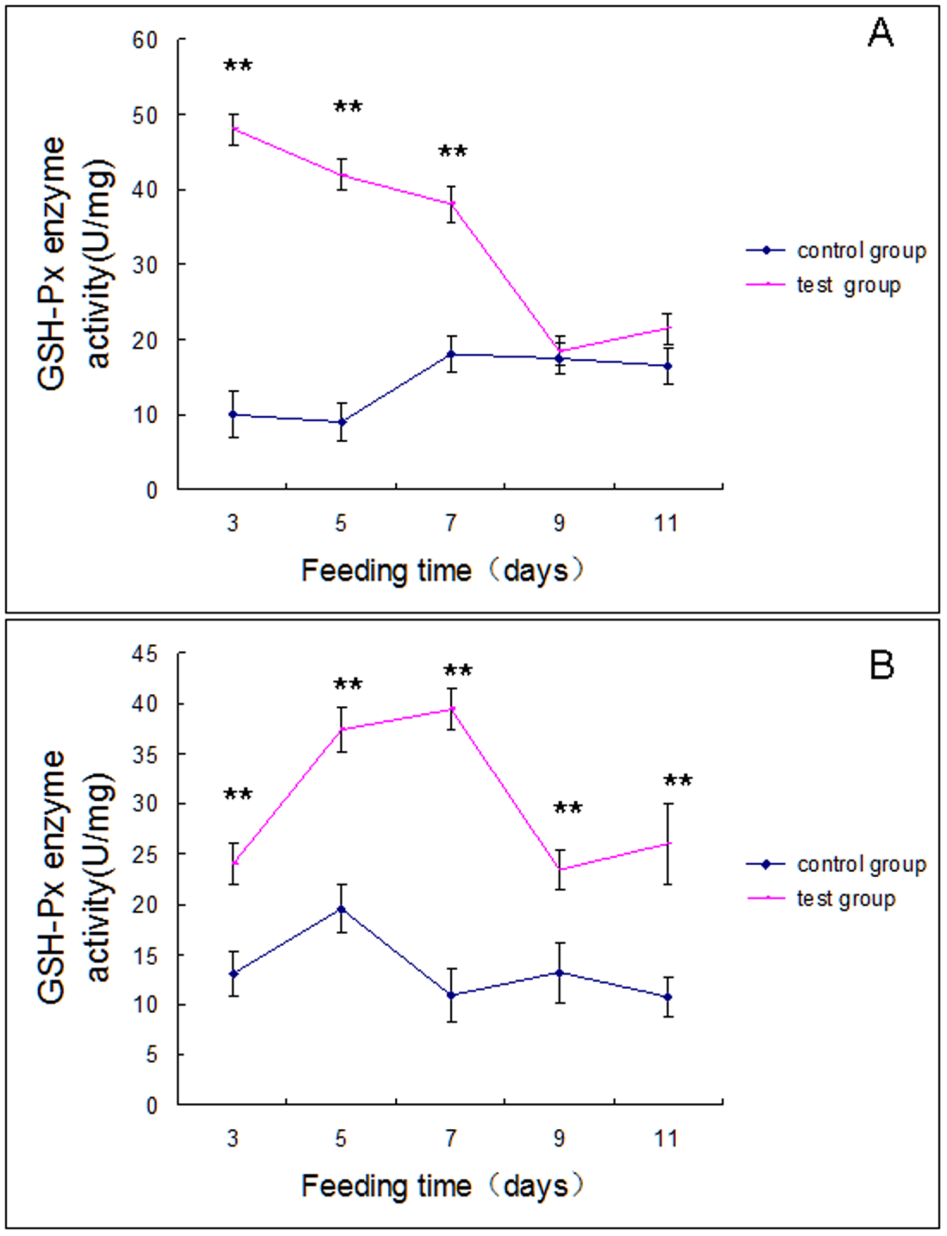
GSH-Px activity in, Panel A, *U. insecticeps* and, Panel B, *P. pseudoannulata* at the indicated times. Each data point reflects the mean ± SE of three biological replicates. The significant differences (*p*<0.05) are indicated by an asterisk. The most significant differences (*p*<0.01) are indicated by two asterisks.

Dietary Cry1Ab protein increased GSH-Px activities in both spider species (Fig. 4). For *U. insecticeps*, GSH-Px activities were about 5-fold higher than control spiders by day 3 of the experimental feeding regimen, then declined to control values by day 9 (Fig. 4A). GSH-Px activities were higher than controls throughout the feeding period in *P. pseudoannulata* (Fig. 4B).

**Figure 5:**
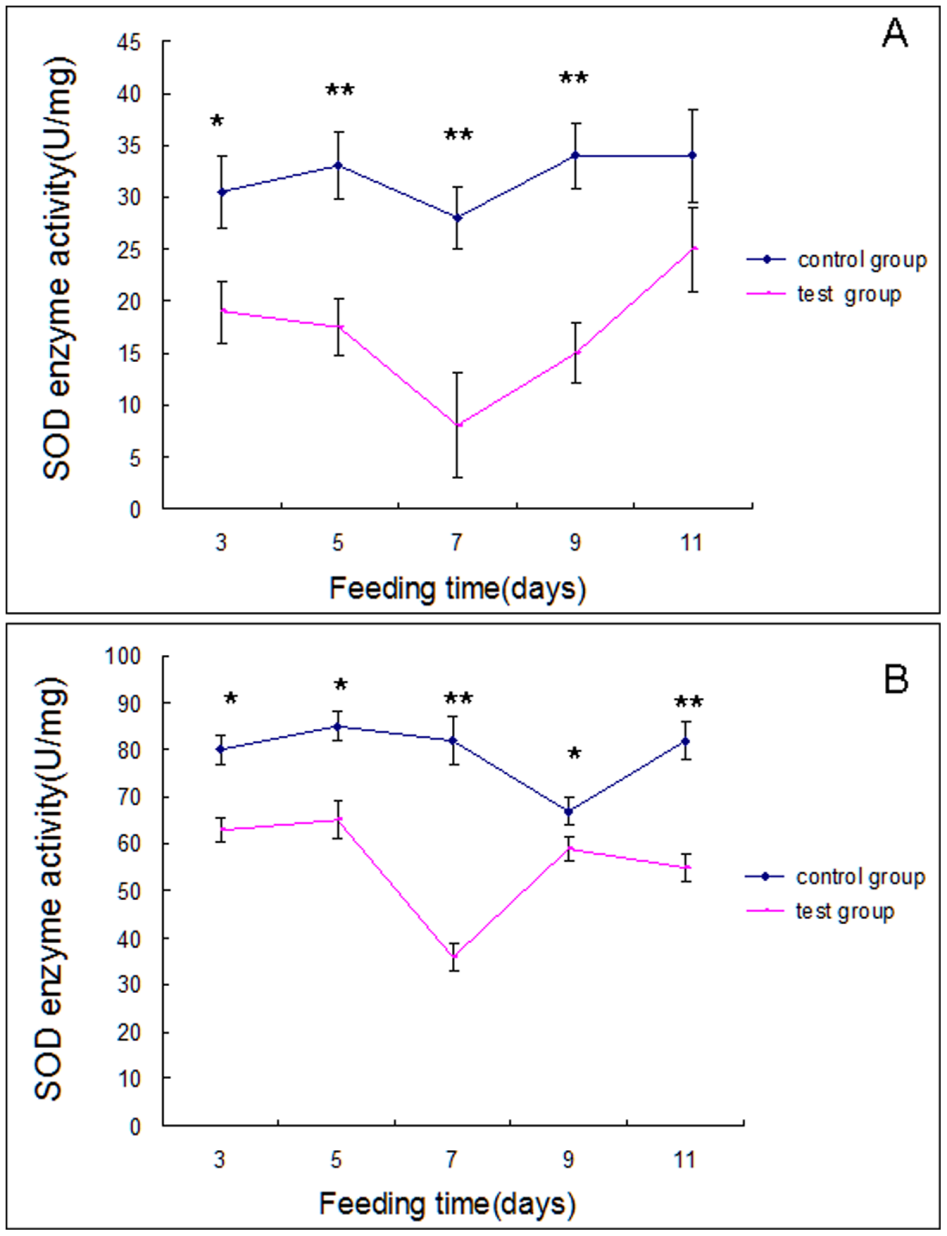
SOD activity in, Panel A, *U. insecticeps* and, Panel B, *P. pseudoannulata* at the indicated times. Each data point reflects the mean ± SE of three biological replicates. The significant differences (*p*<0.05) are indicated by an asterisk. The most significant differences (*p*<0.01) are indicated by two asterisks.

SOD activities were lower, compared to controls, in experimental spiders throughout the feeding experiment, with the exception of day 11 in *U. insecticeps* (Fig. 5). The Y-axis scales differ between Fig. 5A and 5B: *P. pseudoannulata* expressed higher SOD activities in experimentals and controls, compared to *U. insecticeps*.

## Discussion

The data presented in this paper strongly support our hypothesis that intoxication with *Bt* protoxins influences enzyme activities in spiders acting in tri-trophic food webs. Our data show that fruit flies reared on experimental media amended with Cry1Ab protein accumulated Cry1Ab protein from the culture media and that the predatory spiders accumulated Cry1Ab from their experimentally-reared prey. The activities of three enzymes, AChE, GSH-Px and SOD, were influenced, relative to controls, in all experimental treatments. Taken together, these findings bolster the idea that *Bt* protoxins can move through multi-trophic food webs and can influence the biochemistry and physiology of non-target species.

We selected AChE, GSH-Px and SOD for this study on the basis of their physiological significance in arthropod biology. Acetylcholine is a neurotransmitter that expresses its actions through a family of receptors, all ligand-gated ion channels [23]. Some insecticides, the organophosphates and carbamates, act by inhibiting AChE activity, which is necessary for normal physiology of nervous systems. Dietary Cry1Ab protein altered AChE activity in both spider species in this study and may reduce their fitness by interfering with any physiological system based on normal nervous system function, including behavioral functions such as reproductive behavior.

SOD and GSH-Px are anti-oxidant enzymes, responsible for protecting cells and cell components from oxidative damage. Our data show that the presence of Cry1Ab protein in the food stream led to decreased SOD activity in both spider species. This is contrary to the findings of Liu et al. (2010) [24]. These authors maintained rice leafrollers, *Cnaphalocrocis medinalis*, on Cry1Ab-expressing rice for 24 h, then allowed spiders *P. pseudoannulat*a, to consume the leafrollers. This led to increased SOD activity in the spiders. Again, alterations in the activities of these enzymes could be deleterious to long-term fitness of the spiders. In particular, experiments based on the oxidative stress model of aging revealed that overexpressing SOD led to extended lifespans in *Drosophila* [25]. The influence of SOD deficiency, however, may be subtler than a simple effect of accumulating oxidative damage. Rogina and Helfand (2000) showed that SOD-deficient fruit flies experienced acceleration in the normal age-related pattern of expression of a single marker gene, *wingless* [26]. They concluded that the attenuated life span associated with SOD deficiency was due to increased rate of aging, rather than a pathological oxidative process. Nonetheless, SOD function appears to impact lifespans in animals. We note dietary Cry1Ab exerted opposite effects on GSH-Px and SOD, increasing activity of the former and decreasing activity of the latter. In *Drosophila*, an increase in GSH-Px activity is detrimental when combined with a decrease in Sod1 activity, suggesting the two enzymes have opposing functions in vivo [27]. These differences may relate to the separate cellular functions of SOD and GSH-Px. Although superoxide is formed by many reactions within cells, the mitochondrial electron transport chain is a major source of superoxide, that is, it is a normal product of oxygen metabolism and SOD is highly expressed in mitochondria [28]. GSH-Px generally acts to reduce lipid hydroperoxides, most of which form in the lipid fractions of cells. Hence, these two anti-oxidant enzymes largely occur in different fractions of cells. This may relate to the opposite effects of dietary Cry1Ab on SOD and GSH-Px.

It is not clear how dietary Cry1Ab protein influenced the activity of the three enzymes in this study. The changes were seen at the time scale of several days. At this lengthy scale, Cry1Ab protein could act at any level of protein functioning, including expression of genes, post-translation modifications or direct interactions with the proteins. It is also unclear where Cry1Ab exerted its actions. All experiments were performed by homogenizing whole animals, leaving important questions open. Did the fruit flies and spiders actually accumulate Cry1Ab protein, or was the material simply present in the alimentary canals? How much, if any, of Cry1Ab protein moved from the alimentary canals into the body proper? Figures 1 and 2 show that the Cry1Ab is at least partially cleared from the fly and spider bodies after 10 day exposure for the fly and 6 or 7 day exposure for spiders. Is this due to protein metabolism or to simple excretion? As often happens in newly-opened research corridors, our data on the effects of *Bt* protoxins on the biochemistry and physiology of non-target arthropods prompts meaningful new research directions. One of which is to identify other biochemical and physiological functions that are impacted by *Bt* proteins and another is to reconcile the observed effects of dietary Cry1Ab on three enzymes and a large body of literature showing little effects of *Bt* toxin-expressing rice on rice agroecosystems.
